# Phenotyping of field-grown wheat in the UK highlights contribution of light response of photosynthesis and flag leaf longevity to grain yield

**DOI:** 10.1093/jxb/erx169

**Published:** 2017-06-20

**Authors:** Elizabete Carmo-Silva, P John Andralojc, Joanna C Scales, Steven M Driever, Andrew Mead, Tracy Lawson, Christine A Raines, Martin A J Parry

**Affiliations:** 1 Plant Biology and Crop Science, Rothamsted Research, West Common, Harpenden, Hertfordshire, UK; 2 Lancaster Environment Centre, Lancaster University, Bailrigg, Lancaster, UK; 3 School of Biological Sciences, University of Essex, Wivenhoe Park, Colchester, Essex, UK; 4 Centre for Crop Systems Analysis, Wageningen University, Wageningen AK, The Netherlands; 5 Computational and Systems Biology, Rothamsted Research, West Common, Harpenden, Hertfordshire, UK

**Keywords:** CO_2_ assimilation, crop yield, light response, post-anthesis, pre-anthesis, productivity, Rubisco

## Abstract

Improving photosynthesis is a major target for increasing crop yields and ensuring food security. Phenotyping of photosynthesis in the field is critical to understand the limits to crop performance in agricultural settings. Yet, detailed phenotyping of photosynthetic traits is relatively scarce in field-grown wheat, with previous studies focusing on narrow germplasm selections. Flag leaf photosynthetic traits, crop development, and yield traits were compared in 64 field-grown wheat cultivars in the UK. Pre-anthesis and post-anthesis photosynthetic traits correlated significantly and positively with grain yield and harvest index (HI). These traits included net CO_2_ assimilation measured at ambient CO_2_ concentrations and a range of photosynthetic photon flux densities, and traits associated with the light response of photosynthesis. In most cultivars, photosynthesis decreased post-anthesis compared with pre-anthesis, and this was associated with decreased Rubisco activity and abundance. Heritability of photosynthetic traits suggests that phenotypic variation can be used to inform breeding programmes. Specific cultivars were identified with traits relevant to breeding for increased crop yields in the UK: pre-anthesis photosynthesis, post-anthesis photosynthesis, light response of photosynthesis, and Rubisco amounts. The results indicate that flag leaf longevity and operating photosynthetic activity in the canopy can be further exploited to maximize grain filling in UK bread wheat.

## Teaser

Flag leaf longevity and operating photosynthetic rates at ambient CO_2_ and a range of light levels correlated with grain yield of UK field-grown wheat, revealing new targets to enhance crop productivity.

## Introduction

In recent years, wheat yield increases have stagnated in many regions of the world (Hall and Richards, 2013). However, an increase in yields of 70% is needed if we are to meet the projected demand for food by 2050 (Tilman *et al.*, 2011; Ray *et al.*, 2013). The yield potential, namely the yield achieved by a crop grown under optimal water and nutrient supplies and maintained free of pests (Evans, 1993), is the product of the plant biomass and harvest index (HI; the fraction of the biomass allocated to the grain). In most cereals, HI has been optimized through breeding and is now at or close to its theoretical maximum, namely 0.64 for wheat (Austin *et al.*, 1980; Shearman *et al.*, 2005; Foulkes *et al.*, 2007, 2011). The interception of light by wheat canopies has also been improved by breeding and, once the canopy has closed, it is now very efficient (Horton, 2000; Zhu *et al.*, 2010). In contrast, the efficiency of energy conversion into biomass is low, at only about one-third of its theoretical maximum for most crop species, and therefore has potential for significant improvement (Zhu *et al.*, 2008; Ort *et al.*, 2011). In support of this, free-air CO_2_ enrichment (FACE) studies provide compelling evidence that increased photosynthesis translates into greater crop yields (Bernacchi *et al.*, 2006; Long *et al.*, 2006*a*, *b*).

Advances in photosynthesis research in recent years have led to the identification of a number of targets for bioengineering plants with improved carbon assimilation and biomass production (Raines, 2011; Parry *et al.*, 2013). For example, increasing the capacity for ribulose-1,5- bisphosphate (RuBP) regeneration (Driever *et al.*, 2017) or the efficiency and regulation of Rubisco are both plausible strategies to increase photosynthesis and yield, while also improving resource use efficiency (Carmo-Silva *et al.*, 2015). Hence, improving photosynthetic CO_2_ assimilation is a prime target for increasing the productivity of major crops, including wheat (Parry *et al.*, 2011; Simkin *et al.*, 2015). This strategy shows incredible promise, and the technological advances in this area are likely to provide a viable solution towards ensuring food security in the near future.

Existing genotypic variation in photosynthetic efficiency can be exploited by identifying promising cultivars and traits for subsequent integration into breeding programmes aimed at improving crop performance (Fischer and Edmeades, 2010; Lawson *et al.*, 2012). To be effective, this approach requires detailed characterization of the available diversity and an understanding of the underlying processes that determine the observed variation in CO_2_ assimilation rates measured under a range of environmental conditions (Flood *et al.*, 2011; Lawson *et al.*, 2012). Photosynthesis and stomatal conductance have been previously associated with grain yield (GY) in field-grown wheat under irrigated conditions in Mexico (Fischer *et al.*, 1998). In the UK, variation in flag leaf photosynthetic rate and capacity, and in GY, was previously reported for 64 field-grown cultivars (Driever *et al.*, 2014) selected for their Earliness and Resilience for Yield in a Changed Climate (the ERYCC panel; Clarke *et al.*, 2012). The study by Driever *et al.* (2014) did not find a significant correlation between maximum photosynthetic rates measured under optimal conditions at high light and high CO_2_ in flag leaves before ear emergence (Zadoks 4.3–4.5) and GY. Gaju *et al.* (2016), on the other hand, reported a strong positive relationship between flag leaf photosynthetic rates measured in the field at high light and ambient CO_2_ and GY for 15 genotypes, including five UK modern cultivars, five landraces, and five synthetic-derived hexaploid wheat lines, over two field seasons. These observations suggest that the photosynthetic rates observed in the field, rather than maximum photosynthetic capacity, contribute to GY, as previously suggested (Lawson *et al.*, 2012).

The present study reports the results of a follow-up experiment to that reported by Driever *et al.* (2014), using the same wheat cultivars and grown in the subsequent growing season (2013). The objectives specific to this study were to: (i) determine flag leaf photosynthetic performance at two key growth stages, pre-anthesis (Zadoks 4.3–4.5) and post-anthesis (7 d after Zadoks 6.5), enabling evaluation of the impact of net CO_2_ assimilation on GY; (ii) measure the relationship between photosynthetic rate and irradiance (light intensity), enabling assessment of the contribution of photosynthetic light response measured at ambient CO_2_ concentrations, as experienced in the field, to yield; and (iii) determine the contribution of flag leaf Rubisco activity and abundance to the variation in photosynthetic performance.

The results enabled the identification of traits and wheat cultivars with potential for improving wheat productivity and resource use efficiency.

## Materials and methods

### Plant material, experimental design, and growth conditions

The 64 wheat cultivars (*Triticum aestivum* L.) studied were previously selected for their Earliness and Resilience for Yield in a Changed Climate (ERYCC) in a Sustainable Arable LINK project funded by the Department for Environment, Food and Rural Affairs and HGCA (Project LK0992, Adapting Wheat to Global Warming; Clarke *et al.*, 2012). The same project generated genotyping data including the presence/absence of key genes for wheat development and disease resistance (Clarke *et al.*, 2012), which were used herein to investigate possible links with performance in the field. The thousand-grain weight of the seed used for planting (TGW_Planting_) was determined for each cultivar by weighing two subsamples of 500 grains each. The field experiment was planted on 12 December 2012 at the Rothamsted farm (Black Horse field) with three 3 × 1 m plots of each cultivar, giving a total of 192 plots, arranged in an array of 8 rows by 24 columns. Cultivars were randomly allocated to plots following a resolvable alpha design with 8 subblocks, each of 8 plots, per replicate block, with each replicate block of 64 plots being arranged in an array of 8 rows and 8 columns. This design allows for spatial variation across rows and columns to be removed, if present, before assessing for differences between cultivars and estimating heritability.

The agronomic practices adopted were as described before for a trial using the same cultivars (Driever *et al.*, 2014). Fertilizers were applied on: 16 April 2013, 126 days after planting (DAP), 222 kg ha^−1^ DoubleTop^®^ (ammonium sulphate and ammonium nitrate, CF Fertilisers UK Ltd, Ince, UK); 30 April 2013, 140 DAP, 348 kg ha^−1^ Nitram (nitrogen, CF Fertilisers UK Ltd). Fungicides were applied on: 16 May 2013, 156 DAP, 1.25 l ha^−1^ Kingdom^®^ (BASF plc Crop Protection, Cheadle, UK), 1.0 l ha^−1^ Bravo^®^ 500 (Syngenta International AG, Basel, Switzerland); 6 June 2013, 177 DAP, 1.2 l ha^−1^ Ignite^®^ (BASF), 0.4 l ha^−1^ Comet^®^ (BASF); 14 June 2013, 185 DAP, 0.2 l ha^−1^ Cyflamid^®^ (Certis UK, Great Abington, UK); 19 June 2013, 190 DAP, 0.55 l ha^−1^ Cello (Bayer CropScience Ltd, Cambridge, UK), 0.5 l ha^−1^ Corbel^®^ (BASF).

**Fig. 1.**
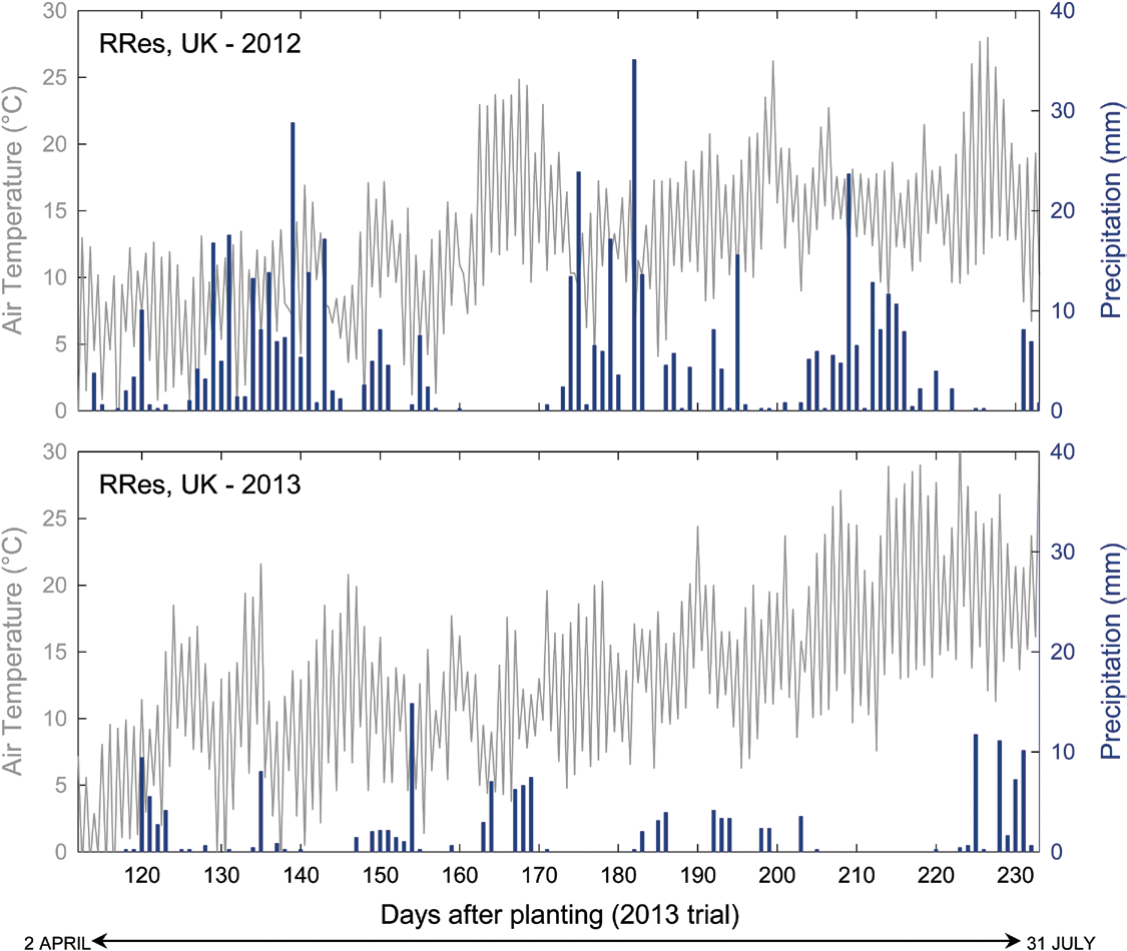
Daily maximum and minimum air temperature and daily precipitation at Rothamsted, Harpenden, UK, from early April to late July in 2012 and 2013. The times when measurements were taken at pre-anthesis (5–14 June 2013; flag leaf fully emerged) and post-anthesis (1–11 July 2013; 7 d after half of the anthers per ear emerged) are indicated for the season of 2013. The corresponding time of the year when measurements were taken in the 2012 trial (Driever *et al.*, 2014) is indicated for comparison (22–30 May 2012; pre-anthesis, flag leaf fully emerged). (This figure is available in colour at *JXB* online.)

The spring of 2013 was relatively cool, but the summer was warm, and rainfall from April through July was considerably lower than in the same period in 2012 (Fig. 1). Solar irradiance and duration from April to July 2013 were relatively high and close to the UK average (Supplementary Table S1 at *JXB* online; Driever *et al.*, 2014). From January to June 2013, maximum and minimum temperatures were below the 30 year average for the UK, but in July and August temperatures were 1–2 °C warmer than the 30 year average.

### Crop agronomic and yield traits

Crop development was monitored throughout the growing cycle. The wheat growth stage was monitored using the Zadoks (Z) scale (Zadoks *et al.*, 1974). The extent of flag leaf senescence in the plots was monitored using the guide provided in Pask *et al.* (2012). Flag leaf longevity (Z4–S5) was estimated from the number of days between emergence (Z4) and 50% senescence (S5). Plant height was measured from the soil surface to the top of the canopy (e.g. tip of the spike) using a ruler. The leaf area index (LAI) was estimated using a canopy analyser (SunScan type SS1 with Sunshine Sensor type BF3; Delta-T Devices Ltd, Cambridge, UK) by determining the fraction of photosynthetically active radiation intercepted by the canopy (Bréda, 2003). Chlorophyll content was estimated with a portable chlorophyll meter (SPAD-502; Konica Minolta, Inc., Tokyo, Japan).

The crop was harvested on 28 August 2013, when all the cultivars had reached physiological maturity and the grain was hard (Z9.1–9.2). Moisture content, total above-ground biomass at 100% dry matter (DM), and grain weight at 85% DM (GY) were determined for each plot as previously described (Driever *et al.*, 2014), using data obtained by the Haldrup plot combine when harvesting all the plants in each plot. The harvest index (HI) was calculated as the ratio between plot grain weight and total above-ground biomass, both at 100% DM.

### Sampling flag leaves for determination of photosynthetic traits

Photosynthetic traits of flag leaves were measured for one representative plant of each of the 192 plots at two developmental stages: pre-anthesis (Z4.3–4.5), shortly after flag leaves had fully emerged; and 7 d post-anthesis (7 d after 50% of the spikes in a plot had reached mid-anthesis, Z6.5). Plant development was monitored on a daily basis from the initiation of booting to mid-anthesis in order to ensure that all cultivars were measured at the same developmental stage. Plants were selected for analysis based on developmental stage, with 21–27 plants from the triplicate plots of 7–9 cultivars being measured each day. Measurements for all replicates of the 64 cultivars, at each growth stage, were completed within a period of 10–11 d, corresponding to 176–185 (pre-anthesis) and 202–212 (post-anthesis) DAP. Flag leaves measured post-anthesis showed signs of physiological leaf spot (Smiley *et al.*, 1993), but were otherwise healthy, showing no visible signs of disease. Flag leaves analysed post-anthesis scored no more than S1 for senescence (10% of the leaf starting to senesce).

Sampling and measurements were performed essentially as previously described (Driever *et al.*, 2014). Shoots were collected from the field before dawn and placed in darkness in a controlled-environment cabinet at 10 °C and 90% relative humidity, to simulate night-time conditions. Prior to gas exchange measurements, while still in darkness, chlorophyll content was estimated with a portable chlorophyll meter (SPAD-502; Konica Minolta) and the maximum quantum efficiency of PSII photochemistry (*F*_v_/*F*_m_) of the dark-adapted leaves was measured with a FluorPen (FP100; Photon Systems Instruments, Ltd, Drasov, Czech Republic). Both SPAD and *F*_v_/*F*_m_ measurements were made at three points along the leaf blade (1/4, 1/2, and 3/4 of the blade length from the adjacent stem; Hamblin *et al.*, 2014).

While the shoots were still in darkness, flag leaf blades were cut under water at the base of the lamina and the cut base placed in a tube containing deionized water, as described by Driever *et al.* (2014). Leaves were then transferred to a controlled-environment cabinet for light adaptation at a photosynthetic photon flux density (PPFD) of 650 μmol m^−2^ s^−1^, 20 °C, and 60% relative humidity for 30–50 min before gas exchange and chlorophyll fluorescence analyses.

After the gas exchange measurements, leaves were transferred to a controlled-environment cabinet for light re-adaptation at a PPFD of 900 μmol m^−2^ s^−1^, 15 °C, and 60% relative humidity for 30–50 min. A sample incorporating the lamina surface used for gas exchange was freeze-clamped (rapidly cooled to the boiling point of liquid N_2_) while still illuminated. Measurement of leaf width of the frozen samples and the width of any gap between the leaf edge and tong perimeter (when these were narrower than the 2.6 cm diameter of the clamping tongs) enabled precise calculation of the sampled area. Samples were subsequently stored at –80 °C until extraction.

Three leaf discs (1.5 cm^2^ total area) were also taken from the same leaf for determination of dry weight and calculation of leaf mass per area (LMA).

### Chlorophyll fluorescence and photosynthetic gas exchange

Infra-red gas analyses (IRGAs) were performed using three LI-6400 XT instruments with integrated leaf chamber fluorometers (LI-COR Biosciences, Lincoln, NE, USA) in parallel. Leaf widths were measured and used to correct gas exchange data for the actual leaf area, on the occasions when the leaf did not fill the chamber completely (i.e. when leaf width was <1.6 cm). Initial conditions in the chambers were: reference CO_2_ concentration (inlet gas), 400 μmol mol^−1^; PPFD, 1800 μmol m^−2^ s^−1^; vapour pressure deficit, ~0.9 kPa; and block temperature, 20 °C. The response of net CO_2_ assimilation (*A*) to the intercellular CO_2_ concentration (*A*/*C*_i_ curve; described by Driever *et al.*, 2014) was followed by measurement of the light response of *A* at 400 μmol CO_2_ mol^−1^ air and decreasing PPFD stepwise from 2000 μmol m^−2^ s^−1^ to 20 μmol m^−2^ s^−1^ (*A*/*Q* or light–response curve) for each leaf. Before every measurement was logged, and as soon as *A* had stabilized for any given set of conditions (~2 min), the reference and sample IRGA signals were matched.

Gas exchange and fluorescence parameters were calculated by the LI-COR OPEN software. The experimental data were modelled using the *A*/*C*_i_ Response Curve Fitting 10.0 and Light Response Curve Fitting 1.0 tools available at http://landflux.org/Tools.php, together with the Rubisco kinetic constants for wheat (Carmo-Silva *et al.*, 2010). The *A*/*C*_i_ curve fitting tool uses model equations and provides parameter estimates as defined by Ethier and Livingston (2004). The light curve fitting tool estimates parameters from a non-rectangular hyperbola (Marshall and Biscoe, 1980; Ögren and Evans, 1993).

### Protein extraction and Rubisco assays

Leaf homogenates were prepared from the samples previously harvested and stored at –80 °C by grinding the leaves at 4 °C with an ice-cold pestle and mortar containing 0.867 ml of 50 mM Bicine-NaOH pH 8.2, 20 mM MgCl_2_, 1 mM EDTA, 2 mM benzamidine, 5 mM ε-aminocaproic acid, 50 mM 2-mercaptoethanol, 10 mM DTT, 1% (v/v) protease inhibitor cocktail (Sigma-Aldrich Co., St Louis, MO, USA), 1 mM phenylmethylsulphonyl fluoride (PMSF; added to the mortar just before grinding), 5% (w/v) polyvinylpolypyrrolidone, and 5% (w/v) acid-washed sand. The homogenate was clarified by centrifugation at 14 700 *g* and 4 °C for 3 min. The supernatant was immediately used to measure the activities and quantity of Rubisco, with two analytical replicates for each measurement.

Rubisco activity was determined by incorporation of ^14^CO_2_ into acid-stable products at 30 °C (Parry *et al.*, 1997). The reaction mixture (final volume 0.5 ml) contained 100 mM Bicine-NaOH pH 8.2, 20 mM MgCl_2_, 10 mM NaH^14^CO_3_ (9.25 kBq μmol^−1^), 2 mM KH_2_PO_4_, and 0.6 mM RuBP. Initial activity assays were started by adding 25 μl of supernatant to the complete assay mixture, while total activity assays were started by adding RuBP to the mixture containing all components (except RuBP) 3 min after adding 25 μl of supernatant, to allow prior carbamylation of Rubisco. Reactions were quenched after 30 s by adding 100 μl of 10 M formic acid. Assay mixtures were dried at 100 °C and the residue re-dissolved in 0.4 ml of deionized water. Acid-stable ^14^C was determined after addition of 4 ml of scintillation cocktail (Ultima Gold, PerkinElmer, Waltham, MA, USA) by liquid scintillation counting (Packard Tri-Carb, PerkinElmer). The Rubisco activation state was calculated from the ratio initial/total activity. Rubisco in 150 μl of the same supernatant was quantified by the [^14^C]CABP [carboxyarabinitol-1, 5-bisphosphate] binding assay (Parry *et al.*, 1997), as described previously (Driever *et al.*, 2014). Total soluble protein content in the supernatants was determined by the method of Bradford (1976).

### Statistical analyses

Initially a linear mixed model was fitted to all measured traits, using the method of residual maximum likelihood (REML), to check for any spatial variability patterns across rows and columns of plots within and across replicate blocks, and to assess for differences due to the three IRGAs used for the photosynthesis measurements. In the absence of any statistically significant additional sources of variability, subsequent analyses assumed that the field trial was arranged following a randomized complete block design. ANOVA was applied to all traits measured (Table 1), taking into account the repeated measurements at the two growth stages, where appropriate, by assuming a split-plot arrangement with cultivar applied at the plot level and growth stage at the subplot level. Mean values were produced for each combination of cultivar and growth stage where the interaction between cultivar and growth stage was significant (*P*<0.05, *F*-test), along with least significant differences (LSDs) for appropriate comparisons at the 5% significance level. No transformations of data were required, with diagnostic plots for the residuals broadly conforming to the assumptions underlying ANOVA. Matrices showing Pearson product–moment correlation coefficients (SigmaPlot 12.0, Systat Software, Inc.) for every pair of measured traits were constructed based on the mean trait values for each cultivar (or cultivar by growth stage combination). Further linear mixed model analyses were applied using the REML method and assuming that the field trial was arranged following a randomized complete block design to estimate the variance component associated with variation between cultivars for each measured trait. Broad sense heritability (*H*^2^) was estimated for each measured trait following the procedure described by Cullis *et al.* (2006), based on the ratio of the between-cultivar variance component and the mean variance of the difference between two cultivar means, as estimated by best linear unbiased predictors (BLUPs), using the results of the linear mixed model analyses. *H*^2^ was also estimated for a limited number of traits measured for the 2012 trial described in Driever *et al.* (2014). Because *H*^2^ is a ratio of variances, it always has a value between 0 and 1 (Wray and Visscher, 2008). *H*^2^ values close to 1 indicate a strong genetic basis for the observed phenotypic variation (highly heritable traits), and values close to 0 indicate low genetic stability and strong environmental (i.e. spatial) control over observed phenotypic variation. All REML and ANOVA applications used GenStat (16th–18th editions, VSN International Ltd).

**Table 1.** Physiological and agronomical traits determined for 64 field-grown wheat cultivars (ERYCC panel) in 2013 in the UK

Trait	Description
TGW_Planting_	Thousand-grain weight (g), dry weight of 1000 grains, determined for seed used for planting
Biomass	Total above-ground biomass (t ha^−1^), straw and grain dry weight, determined by harvest of whole plot
Straw	Straw biomass (t ha^−1^), straw dry weight, determined by harvest of whole plot
GY	Grain yield (t ha^−1^), grain weight at 85% dry matter, determined by harvest of whole plot
HI	Harvest index (kg grain kg^−1^ biomass), grain dry weight as a fraction of total above-ground biomass
Z4, Z6.5	Time (days after planting, DAP) at which Zadoks stages 4 or 6.5 were reached
S5, S10	Time (days after planting, DAP) at which senescence scores S5 and S10 were reached
LAI	Leaf area index (relative units), measured at four locations per plot
Height	Mean plant height (cm), measured for four representative plants per plot
SPAD_Plot_	Mean SPAD (leaf chlorophyll) of flag leaves, measured for four plants per plot
SPAD_Leaf_	Mean SPAD (leaf chlorophyll) of leaf used in gas exchange analysis, measured at three points per leaf
*F*_v_/*F*_m_,_Dark_	Mean quantum yield (*F*_v_/*F*_m_) of leaf used in gas exchange analysis, taken while dark-adapted (FluorPen)
*F*_v_'/*F*_m_'	Maximum quantum efficiency of PSII in the light (relative units)
LMA	Leaf mass per area (g m^−2^), determined with three leaf disks from the leaf used in gas exchange
*A*_Q250_	Net CO_2_ assimilation rate (μmol m^−2^ s^−1^) at PPFD 250 μmol photons m^−2^ s^−1^ and 400 μmol mol^−1^ CO_2_
*A*_Q500_	Net CO_2_ assimilation rate (μmol m^−2^ s^−1^) at PPFD 500 μmol photons m^−2^ s^−1^ and 400 μmol mol^−1^ CO_2_
*A*_Q1000_	Net CO_2_ assimilation rate (μmol m^−2^ s^−1^) at PPFD 1000 μmol photons m^−2^ s^−1^ and 400 μmol mol^−1^ CO_2_
*A*_Q1800_	Net CO_2_ assimilation rate (μmol m^−2^ s^−1^) at PPFD 1800 μmol photons m^−2^ s^−1^ and 400 μmol mol^−1^ CO_2_
*A*_max_	Maximum net CO_2_ assimilation rate (μmol m^−2^ s^−1^) at PPFD 1800 μmol m^−2^ s^−1^ and 1200 μmol mol^−1^ CO_2_
*g*_*s*_	Stomatal conductance to water vapour (mol m^−2^ s^−1^) at PPFD 1800 μmol m^−2^ s^−1^ and 400 μmol mol^−1^ CO_2_
*T*_leaf_	Leaf temperature (°C), measured by thermocouple
*V*_cmax_	Maximum carboxylation activity of Rubisco (μmol CO_2_ m^−2^ s^−1^), estimated by *A*/*C*_i_ curve fitting
*J*_max_	Maximum electron transport rate (μmol electrons m^−2^ s^−1^), estimated by *A*/*C*_i_ curve fitting
*J*_max_/*V*_cmax_	Ratio between *J*_max_ and *V*_cmax_ (μmol electrons μmol^−1^ CO_2_)
*C*_i(trans)_	*C*_i_ at which *A* transitions from Rubisco to RuBP regeneration limited (μmol CO_2_ mol^−1^ air)
*C*_i_–*C*_i(trans)_	Difference between *C*_i_ at PPFD 1800 μmol m^−2^ s^−1^ and 400 μmol mol^−1^ CO_2_ and *C*_i(trans)_
*R*_d(*A/Q*)_	Dark respiration (μmol m^−2^ s^−1^), estimated by light curve fitting
φ _(*A*/*Q*)_	Apparent quantum yield (mol CO_2_ mol^−1^ photons), estimated by light curve fitting
θ _(A/*Q*)_	Curvature of the light response of net CO_2_ assimilation (relative units), estimated by light curve fitting
LCP_(*A*/*Q*)_	Light compensation point (μmol photons m^−2^ s^−1^), estimated by light curve fitting
*A*_sat(*A/Q*)_	Net CO_2_ assimilation rate (μmol m^−2^ s^−1^) at saturating PPFD and 400 μmol mol^−1^ CO_2_, estimated by light curve fitting
R_I/T_	Rubisco activation (initial/total activity, relative units)
R_Amt_	Rubisco amount per unit leaf area (g m^−2^)
TSP	Total soluble protein per unit leaf area (g m^−2^)
R/TSP	Rubisco amount relative to total soluble protein (mg mg^−1^)
R_Initial_	Rubisco initial activity (μmol CO_2_ fixed m^−2^ s^−1^), on a leaf area basis
R_Total_	Rubisco total activity (μmol CO_2_ fixed m^−2^ s^−1^), on a leaf area basis

## Results

### Flag leaf longevity and photosynthetic activity contribute to grain yield

**Fig. 2.**
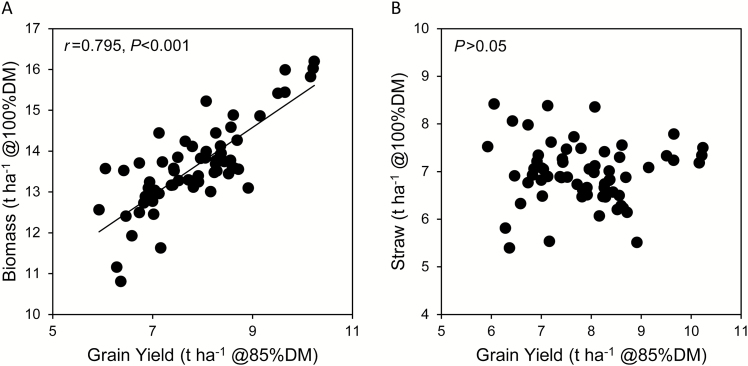
The relationship between plant above-ground biomass and grain yield. The total above-ground biomass correlated positively with grain yield (A), but the straw dry matter did not (B). Values are adjusted means for three separate plots per cultivar.

**Fig. 3.**
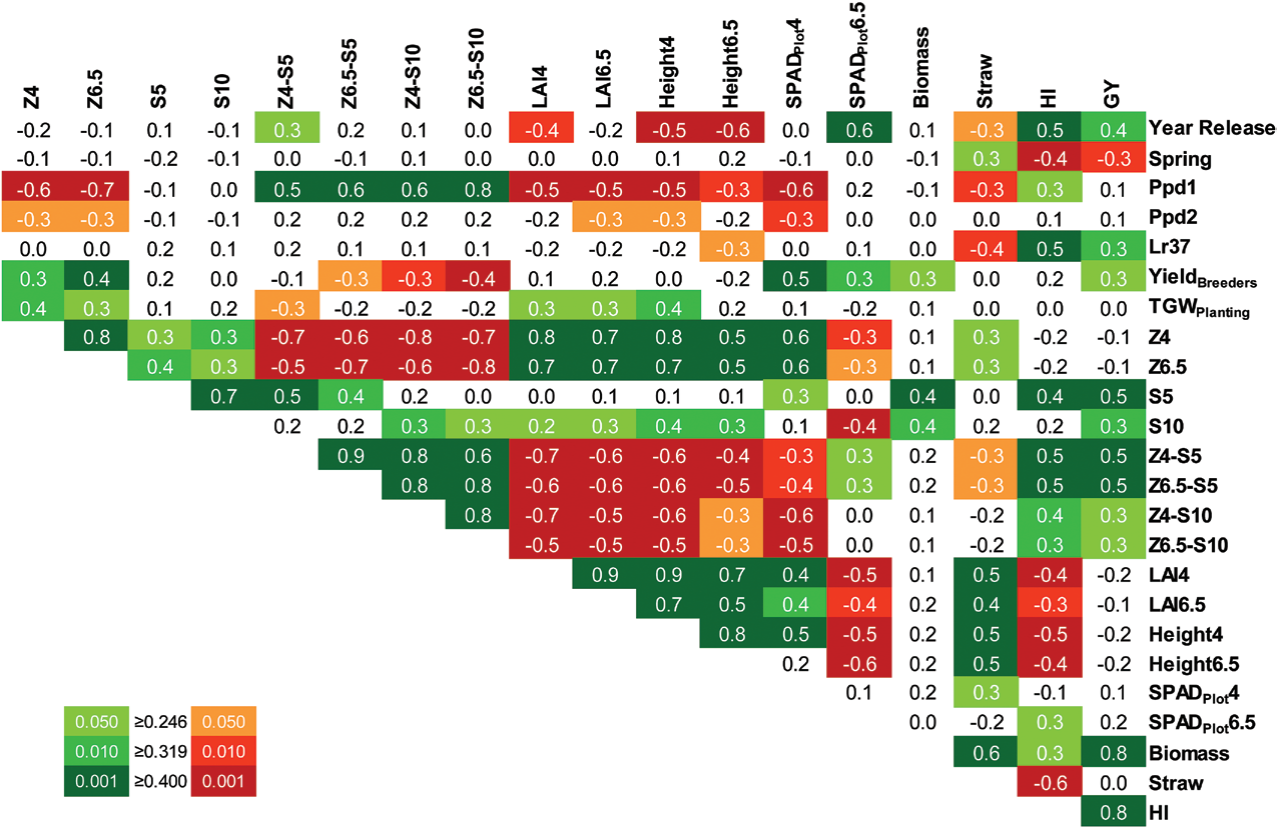
Correlation matrix showing the significance of linear correlations between paired mean values of genetic, physiological, and agronomical traits from 64 field-grown wheat cultivars. Numbers are Pearson product–moment correlation coefficients (*r*, df=62) and increasingly significant correlations (*P*≤0.05, *P*≤0.01, and *P*≤0.001) are indicated by increasingly darker shading. Year of cultivar release, spring growth habit (spring versus winter based on flowering response to temperature), presence of genes *Ppd1*, *Ppd2*, and *Lr37*; yield data over multiple sites and years (Yield_Breeders_) were kindly supplied by the authors of a previous study (Clarke *et al.*, 2012). Measured traits are described in Table 1. (This figure is available in colour at *JXB* online.)

**Fig. 4.**
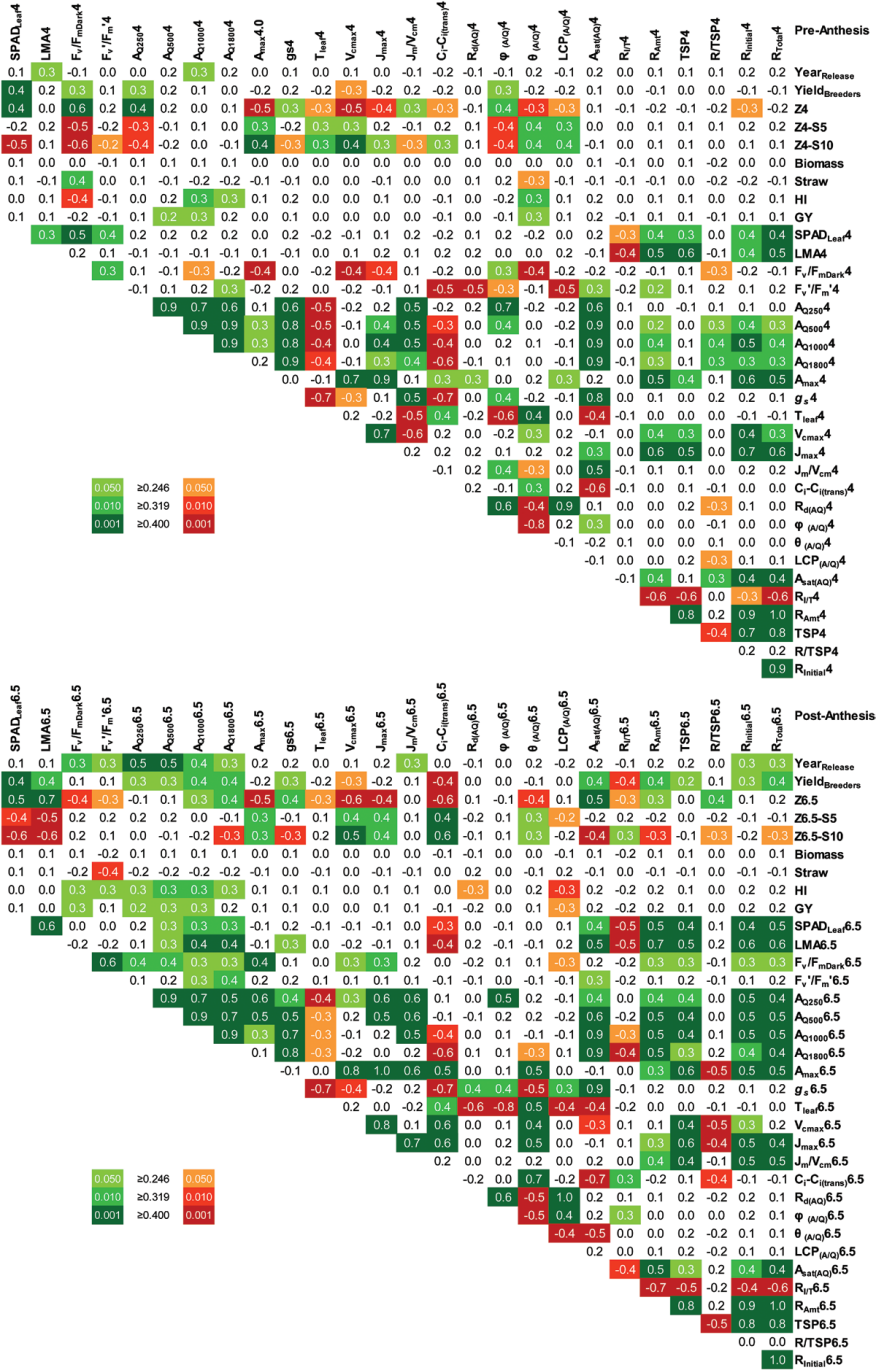
Correlation matrix showing the significance of linear correlations between paired mean values of photosynthetic and yield traits from 64 field-grown wheat cultivars. Numbers are Pearson product–moment correlation coefficients (*r*, df=62) and increasingly significant correlations (*P*≤0.05, *P*≤0.01, and *P*≤0.001) are indicated by increasingly darker shading. Year of cultivar release and yield data over multiple sites and years (Yield_Breeders_) were kindly supplied by the authors of a previous study (Clarke *et al.*, 2012). Measured traits are described in Table 1. (This figure is available in colour at *JXB* online.)

**Fig. 5.**
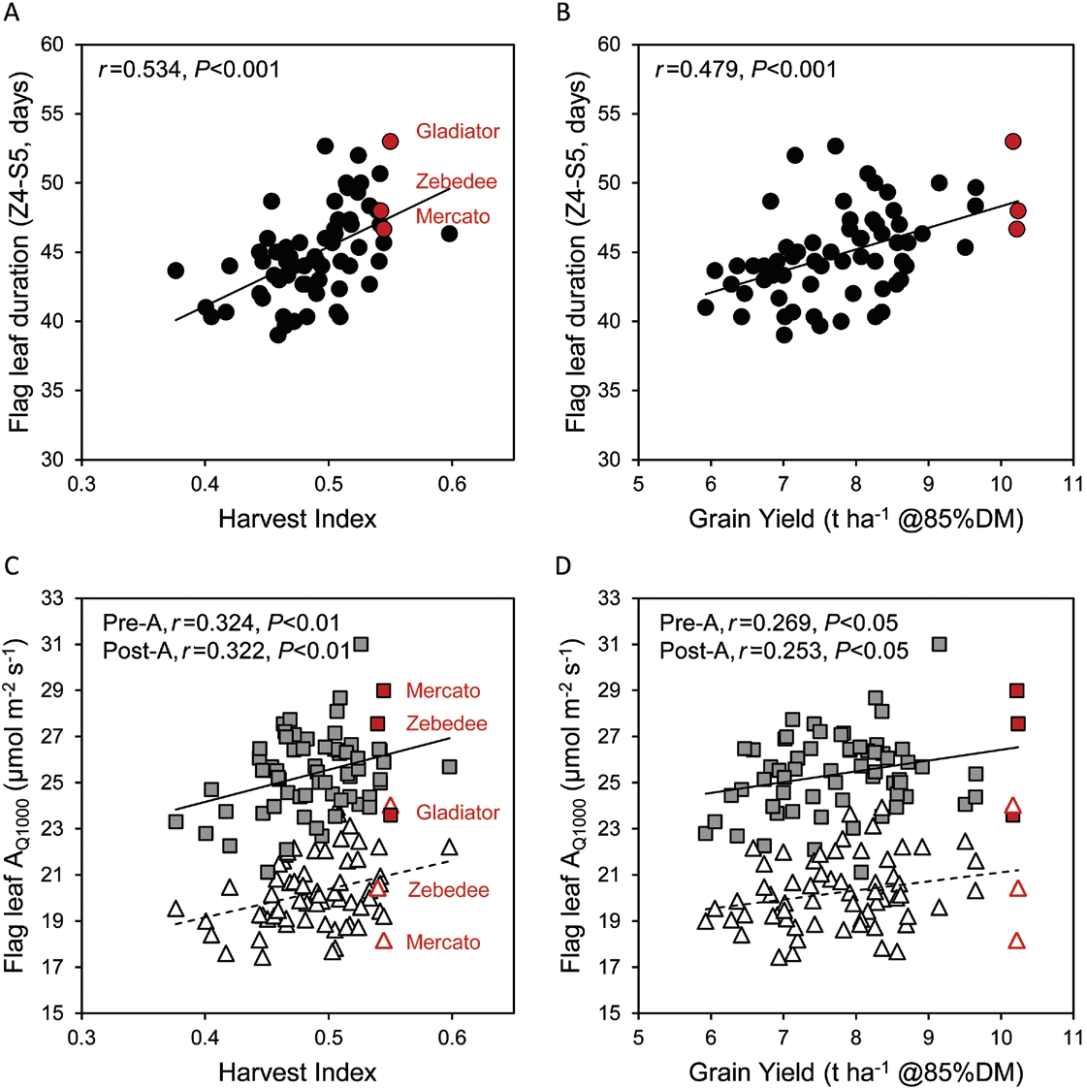
The relationship between flag leaf longevity (A, B) or photosynthetic activity (C, D) and harvest index (A, C) or grain yield (B, D) in 64 field-grown wheat cultivars. Flag leaf longevity was the time between flag leaf emergence and 50% senescence (Z4–S5) and correlated positively with harvest index (A) and grain yield (B). Net CO_2_ assimilation rates of pre- and post-anthesis flag leaves at 400 μmol mol^−1^ CO_2_ and 1000 μmol photons m^−2^ s^−1^ (*A*_Q1000_) correlated positively with harvest index (C) and grain yield (D). The highest yielding cultivars Mercato, Zebedee, and Gladiator are identified. Data points are adjusted means for three separate plots per cultivar. (This figure is available in colour at *JXB* online.)

The GY of the 64 wheat cultivars in 2013 varied between 5.9 t ha^−1^ and 10.2 t ha^−1^, with an overall mean of 7.9 t ha^−1^, and was closely associated with the HI (*r*=0.798, df=62, *P*<0.001), the latter ranging from 0.38 to 0.60 and an overall mean of 0.49. The total above-ground biomass correlated strongly and positively with GY (Fig. 2), but did not correlate with agronomic or photosynthetic traits (Figs 3, 4). On the other hand, the straw DM, reflecting investment in leaves and stems, did not correlate with GY (Fig. 2), correlated negatively with HI, and correlated positively with time to reach spike emergence (Z4 and Z6.5), LAI, and plant height (Fig. 3). In other words, plants taking longer to reach Z4 and Z6.5 had greater LAI and plant height at these growth stages and were greener pre-anthesis, but not post-anthesis. The time to reach Z4 and Z6.5 did not correlate with GY, while the time to reach senescence (S5) and the time between Z4 and Z6.5 and senescence scores S5 and S10 correlated positively with both HI and GY (Figs 3, 5). These results suggest that plant biomass prior to emergence of the flag leaf does not contribute to GY, while the longevity of the flag leaf does.

Flag leaf photosynthesis measured both pre- and post-anthesis at current ambient CO_2_ and a PPFD of 1000 μmol m^−2^ s^−1^ (*A*_Q1000_) correlated positively with both HI and GY (Fig. 5). Pre-anthesis *A*_Q1000_ varied between 21.1 μmol m^−2^ s^−1^ and 31.0 μmol m^−2^ s^−1^, with an overall mean of 25.4 μmol m^−2^ s^−1^, and was highly correlated with stomatal conductance to water vapour (*g*_*s*_). For most cultivars, *A*_Q1000_ decreased post-anthesis compared with pre-anthesis, but some cultivars maintained high rates of photosynthesis at both growth stages. Post-anthesis *A*_Q1000_ ranged from 17.4 μmol m^−2^ s^−1^ to 24.0 μmol m^−2^ s^−1^, with an overall mean of 20.3 μmol m^−2^ s^−1^. The cultivars Gladiator, Battalion, and Brompton had the highest *A*_Q1000_ values post-anthesis, showing no significant difference from the rates measured pre-anthesis (*P*>0.05). The maximum net CO_2_ assimilation rate at saturating PPFD and high CO_2_ (*A*_max_) also decreased, from an overall mean of 50.9 μmol m^−2^ s^−1^ pre-anthesis to 39.0 μmol m^−2^ s^−1^ post-anthesis; however, *A*_max_ was not correlated to HI or GY (Fig. 4), in agreement with the results reported by Driever *et al.* (2014). These results indicate that the operational photosynthetic rate, under prevailing environmental conditions, rather than the maximum photosynthetic capacity of the flag leaf, contributes to grain filling and might be a good predictor of grain yield.

The contribution of the light response of flag leaf photosynthesis to grain filling was evidenced by positive correlations between several photosynthetic traits and HI, as well as GY (Fig. 4). Overall mean values for *A*_Q250_, *A*_Q500_, *A*_Q1000_, and *A*_Q1800_ were 11.4, 19.2, 25.4, and 27.9 μmol m^−2^ s^−1^ pre-anthesis and 10.2, 16.0, 20.3, and 21.9 μmol m^−2^ s^−1^ post-anthesis. Pre-anthesis *A*_Q1000_, *A*_Q1800_, and the curvature of the light–response curve [θ_(*A*/*Q*)_] correlated positively with HI. Post-anthesis *A*_Q250_, *A*_Q500_, *A*_Q1000_, *A*_Q1800_, and *F*_v_′/*F*_m_′ correlated positively, and the light compensation point correlated negatively with HI. Most of these photosynthetic traits also correlated significantly with GY, albeit less strongly than with HI. *g*_*s*_ correlated positively, and leaf temperature (*T*_leaf_) correlated negatively, with photosynthetic rates measured pre- and post-anthesis. However, neither *g*_*s*_ nor *T*_leaf_ correlated significantly with HI or GY (Fig. 4). Taken together, the results suggest that the ability to maintain high operating photosynthetic activity throughout the lifespan of the flag leaf contributes to GY.

### Rubisco activity and abundance contribute to high photosynthetic activity throughout grain filling

**Fig. 6.**
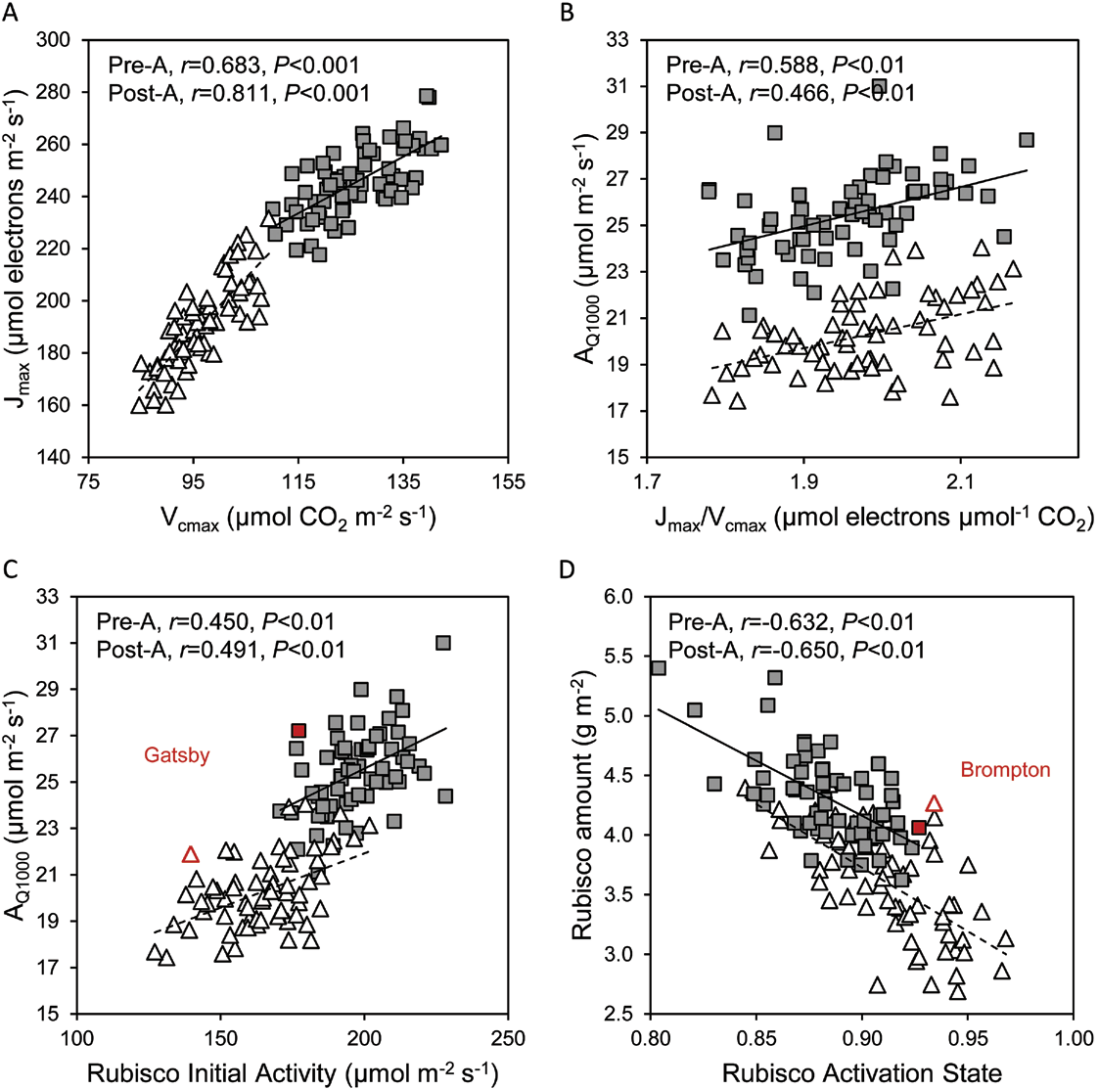
The relationship between photosynthetic traits in flag leaves of 64 field-grown wheat cultivars. The maximum electron transport rate (*J*_max_) and the maximum carboxylation activity of Rubisco (*V*_cmax_) were highly correlated and both decreased post-anthesis compared with pre-anthesis (A). Net CO_2_ assimilation rates of pre- and post-anthesis flag leaves at 400 μmol mol^−1^ CO_2_ and 1000 μmol photons m^−2^ s^−1^ (*A*_Q1000_) correlated positively with the ratio *J*_max_/*V*_cmax_ (B) and with the Rubisco initial activity per unit leaf area (C). Rubisco amount and activation state correlated negatively (D). Symbols: filled, pre-anthesis (solid lines); open, post-anthesis (dashed lines). Cultivars that stood out (Gatsby and Brompton) are identified. Data points are adjusted means for three separate plots per cultivar. (This figure is available in colour at *JXB* online.)

Absolute values of the maximum electron transport rate (*J*_max_) and maximum carboxylation capacity (*V*_cmax_) decreased post-anthesis (Fig. 6A), with the decrease being slightly more pronounced for *V*_cmax_, resulting in an overall mean *J*_max_/*V*_cmax_ ratio of 1.95 μmol electrons μmol^−1^ CO_2_ and 1.98 μmol electrons μmol^−1^ CO_2_ pre- and post-anthesis, respectively (*P*<0.001). This marginal difference suggests that maintenance of a fine balance between the capacity to carboxylate and to regenerate the CO_2_ acceptor, RuBP, was necessary even after anthesis when leaf protein (including elements of the photosynthetic machinery) would have been in the process of re-allocation to the developing grain (Feller *et al.*, 2008). The photosynthetic capacity (*A*_max_) was highly correlated with the electron transport and carboxylation capacities (*J*_max_ and *V*_cmax_), as well as with the amount of total soluble protein and the amount of Rubisco in the leaves (Fig. 4). The flag LMA, which reflects the concentration of leaf components per leaf area, and the greenness of the leaf, as indicated by the chlorophyll SPAD measurement, also correlated positively with the total soluble protein content and with Rubisco activities and amount per leaf area (Fig. 4). These correlations were more significant post-anthesis. Furthermore, post-anthesis LMA and SPAD_Leaf_ also correlated positively with net CO_2_ assimilation at ambient CO_2_ and over a range of light intensities.

Flag leaf photosynthetic rates across a range of PPFDs was positively correlated with *J*_max_/*V*_cmax_ (Figs 4, 6B) and with the amount and activity of Rubisco, pre-anthesis and, more markedly, post-anthesis (Figs 4, 6C). The *C*_i_ at which the limitation of photosynthesis transitioned from RuBP carboxylation to RuBP regeneration [*C*_i(trans)_] was always higher than the *C*_i_ at ambient levels of CO_2_ and a PPFD of 1800 μmol m^−2^ s^−1^ (mean *C*_i_ values were 210 μmol mol^−1^ pre-anthesis and 232 μmol mol^−1^ post-anthesis). Thus, under field conditions, flag leaf photosynthetic rates appeared to be limited by the activity of RuBP carboxylation (i.e. Rubisco). In this context, Gatsby stood out by its relatively high photosynthetic rates in relation to Rubisco activity (Fig. 6C) both pre- and post-anthesis.

A strong negative correlation between the amount of Rubisco and the Rubisco activation state was evident both pre- and post-anthesis (Fig. 6D). For most cultivars, the amount of Rubisco decreased markedly post-anthesis while the corresponding Rubisco activation states increased. Even so, a few cultivars maintained their post-anthesis Rubisco content close to the corresponding pre-anthesis levels. The cultivar Brompton, for instance, had very similar amounts of flag leaf Rubisco pre- and post-anthesis, and a relatively high Rubisco activation state of ~93% at both growth stages (Fig. 6D).

### Photosynthetic traits are heritable

Broad-sense heritability (*H*^2^) of traits indicated a strong genetic basis for differences observed in HI and, to a lesser extent, GY in 2013 (Table 2). Straw and, particularly, total above-ground biomass were less heritable, indicating a stronger spatial influence over these traits. In contrast, in 2012, biomass, straw, GY, and HI were all highly heritable. Comparison of *H*^2^ for each trait over the 2 years suggests stronger genetic determination of HI, with biomass and final GYs being more dependent on the interaction between genotypes and the surrounding environment. Moreover, the lower *H*^2^ obtained for GY in 2013 compared with 2012 reflects a stronger impact of environment in 2013 (Mackay *et al.*, 2011).

**Table 2.** Broad-sense heritability (*H*^2^) of yield, agronomic, and photosynthetic traits in UK wheat measured pre- and post-anthesis Values obtained for a limited number of traits measured for the 2012 trial (Driever *et al.*, 2014) are provided for comparative purposes

2013			2012	
Yield traits	*H* ^2^		Yield traits	*H* ^2^
Biomass	0.279		Biomass	0.819
Straw	0.471		Straw	0.725
GY	0.576		GY	0.892
HI	0.765		HI	0.710
**Agronomic trait**	***H*** ^**2**^ **pre-anthesis**	***H*** ^**2**^ **post-anthesis**	**Agronomic trait**	***H*** ^**2**^ **pre-anthesis**
Z4–S5		0.667	Z4–S5	–
Z4; Z6.5	0.933	0.966	Z4; Z6.5	–
LAI	0.838	0.860	LAI	0.758
Height	0.892	0.883	Height	–
**Photosynthetic trait**	***H*** ^**2**^ **pre-anthesis**	***H*** ^**2**^ **post-anthesis**	**Photosynthetic trait**	***H*** ^**2**^ **pre-anthesis**
*A*_Q250_	0.464	0.441	*A*_Q250_	–
*A*_Q500_	0.656	0.314	*A*_Q500_	–
*A*_Q1000_	0.721	0.319	*A*_Q1000_	–
*A*_Q1800_	0.709	0.342	*A*_Q1800_	0.713
*A*_max_	0.593	0.544	*A*_max_	0.498
*g*_*s*_	0.764	0.513	*g*_*s*_	–
*F*_v_'/*F*_m_'	0.643	0.639	*F*_v_'/*F*_m_'	–
θ _(A/Q)_	0.429	0.646	θ _(A/Q)_	–
LCP_(A/Q)_	0.267	0.757	LCP_(A/Q)_	–
*A*_sat(A/Q)_	0.730	0.520	*A*_sat(A/Q)_	–
R/TSP	0.448	0.644	R/TSP	–
R_Amt_	0.535	--	R_Amt_	0.524

Flag leaf longevity (Z4–S5), the time to reach Z4 and Z6.5, the LAI, and plant height were also highly heritable, both pre- and post-anthesis, while photosynthetic traits tended to be more heritable either pre-anthesis (net CO_2_ assimilation rates and stomatal conductance) or post-anthesis (light response parameters and the amount of Rubisco relative to total soluble protein). *H*^2^ was estimated for a few photosynthetic traits measured pre-anthesis in the preceding field trial of 2012 (Driever *et al.*, 2014). The remarkable similarity between the *H*^2^ estimated for *A*_Q1800_, *A*_max_, and Rubisco amounts pre-anthesis corroborates the estimates for the single year trail of 2013 and indicates a strong genetic basis for photosynthetic traits in UK wheat cultivars.

Multiple pairwise correlation analysis was used to evaluate the impact of potentially interacting factors and validate the results observed for a field trial conducted over a single year at a single location. Both GY and HI correlated with year of cultivar release (Fig. 3), reflecting crop improvement through breeding efforts over the past decades. Moreover, GY correlated with the weighted mean yield for the 64 cultivar ERYCC panel over many years and locations (Yield_Breeders_; Clarke *et al.*, 2012). However, the relatively poor correlation coefficient between GY and Yield_Breeders_ indicates that the cultivars attaining the highest yields in the 2013 field trial did not fully coincide with those attaining the highest yields across multiple years and locations.

On account of prevailing weather conditions, the trial was planted late (12 December) for winter wheat (which requires vernalization over the autumn and winter months for optimal yields) with optimal planting dates before 25 September. Late planting can lead to spring cultivars and cultivars that are photoperiod insensitive (presence of *Ppd1* and *Ppd2* genes; Scarth *et al.*, 1985) performing better than expected in a winter wheat trial. However, a negative correlation between spring habit and GY was observed, suggesting that cultivars across the panel were similarly influenced by the late planting. The presence of photoperiod insensitivity genes was negatively correlated with the time at which plants reached Z4 or Z6.5 (Fig. 3), indicating faster development of these cultivars. However, there was no correlation with timing of senescence or GY, indicating that late planting did not have a great impact on the yield differences between cultivars.

The seed used for planting the trial was obtained from the field trial of the previous season (Driever *et al.*, 2014). Since the summer of the previous season was very wet and the prevalence of fungal pathogens within the UK was widespread, this may have impacted on seed quality and subsequent crop development in 2013. The thousand-grain weight of seed used for planting (TGW_Planting_) correlated with some subsequent plant developmental traits, such as LAI, but not with biomass or GY (Fig. 3), suggesting that seed vigour was not a major factor determining growth or grain production by the different cultivars. Although leaves did not show signs of disease during measurements, the significant correlation between presence of a gene that confers resistance to leaf rust (*Lr37*; Bariana and McIntosh, 1993) and both GY and HI suggests that disease in the latter stages of crop development may have had a role in determining GY. It is also noteworthy that 2013 was characterized by limited rainfall during grain filling (Fig. 1). Since stress factors can accelerate crop senescence (e.g. Lobell *et al.*, 2012), the above factors could have contributed to decrease flag leaf longevity (Z4–S5) and GY.

## Discussion

Genotypic variation in flag leaf photosynthetic traits correlated with wheat yield traits and can be used to inform wheat breeding programmes aimed at improving wheat productivity. The responses of net CO_2_ assimilation to varying CO_2_ and PPFD were measured pre- and post-anthesis, and examined with reference to above-ground biomass, GY, agronomic traits, leaf structural characteristics, and Rubisco activity and abundance in 64 field-grown wheat cultivars of the ERYCC panel. Our results suggest that flag leaf longevity and photosynthetic efficiency beyond anthesis contribute to grain filling in UK-adapted wheat germplasm. Rubisco amounts in the flag leaves decreased post-anthesis compared with pre-anthesis, while Rubisco activity was up-regulated and correlated with photosynthetic traits, especially post-anthesis. Many of the photosynthetic component traits presented genetic variation and were shown to be consistently heritable and therefore amenable to phenotypic selection, as required for breeding superior wheat cultivars (Poland *et al.*, 2012; Hill *et al.*, 2013; Poland, 2015).

The negative correlation between Rubisco abundance and activation state (Fig. 6D) suggests that a small decrease in Rubisco protein could be ameliorated by a corresponding increase in activation state, leaving overall activity unchanged. This observation is in agreement with studies using transgenic plants, where decreased Rubisco did not affect photosynthesis due to increased activation state (Stitt and Schulze, 1994). The *A*/*C*_i_ response curves suggested that CO_2_ assimilation was mostly limited by the activity of Rubisco at high PPFD and ambient CO_2_ levels. At lower PPFD levels, which frequently prevail in the UK, net CO_2_ assimilation tends to be limited by the regeneration of the Rubisco co-substrate, RuBP (von Caemmerer, 2000). Reducing the Rubisco content of wheat leaves would enable a higher allocation of N to enzymes of the Calvin cycle, such as sedoheptulose-1,7-bisphosphatase, that are currently rate limiting to RuBP regeneration (Raines, 2006; Zhu *et al.*, 2007), with potential benefits for photosynthetic and resource use efficiencies. Moreover, this N re-allocation and the resulting increases in RuBP regeneration are likely to become more beneficial as CO_2_ concentration in the atmosphere increases as a result of climate change.

Pre-anthesis flag leaf CO_2_ assimilation at ambient CO_2_ and 1000–1800 μmol photons m^−2^ s^−1^ (*A*_Q1000_, *A*_Q1800_) as well as the curvature of the light–response curve of photosynthesis (θ) correlated positively with HI and, less strongly, with GY. Photosynthetic rate is limited by CO_2_ diffusion to the chloroplast (stomatal and mesophyll conductance), by the electron transport rate and regeneration of RuBP (*J*) at low PPFD, and by the activity of Rubisco at high PPFD. The higher the value of the photosynthesis light–response curvature (θ), the lower is the PPFD at which the transition to a Rubisco limitation of photosynthesis occurs (Ögren and Evans, 1993). In other words, the positive correlation between θ and both HI and GY pre-anthesis indicates that, in high-yielding cultivars, photosynthetic rates are more likely to be limited by Rubisco activity (RuBP carboxylation) than *J* (electron transport rate and RuBP regeneration), over the wide range of PPFDs observed in the field.

A meta-analysis of photosynthetic responses to CO_2_ (*A*/*C*_i_ data) from 109 C_3_ plant species first highlighted the linear relationship that characterizes the close functional balance in the allocation of resources between *V*_cmax_ (RuBP carboxylation) and *J*_max_ (RuBP regeneration), in spite of widely differing photosynthetic capacities among the chosen species (Wullschleger, 1993). Remarkably, the mean *J*_max_/*V*_cmax_ reported (1.97 μmol electrons μmol^−1^ CO_2_) was very close to that obtained using the present data (Fig. 6A). Maintenance of the *J*_max_/*V*_cmax_ ratio beyond anthesis demonstrates the functional balance of photosynthetic components underpinning continued CO_2_ assimilation during the gradual re-allocation of leaf protein to the developing grain.

A strong positive correlation between pre-anthesis photosynthesis and GY was recently reported for 15 field-grown wheat genotypes including UK modern cultivars, landraces, and synthetic derivatives grown in the UK over two field seasons (Gaju *et al.*, 2016). One could argue that the correlations observed in the present study between pre- and post-anthesis flag leaf photosynthesis and GY or HI, albeit significant, were not very strong. Importantly, photosynthetic traits were measured in plants under near ideal conditions, with plentiful hydration and uniform leaf illumination, unlike the variable conditions prevailing during growth in the field (Lawson *et al.*, 2012), which are likely to have differentially affected the measured responses of the diverse cultivars across the panel. The significant correlations between pre- and post-anthesis photosynthesis and HI/GY support that breeding for wheat yield in the UK has led to co-selection of both high photosynthetic potential and partitioning of assimilates to the grain. A similar conclusion was reached for eight field-grown spring wheat cultivars under irrigated conditions in Mexico over three growing seasons (Fischer *et al.*, 1998), with increased photosynthesis being mostly driven by increased stomatal conductance under those conditions, which impacts on both CO_2_ diffusion and leaf temperature.

As the flag leaves aged, decreased post-anthesis photosynthetic rates were accompanied by decreased chlorophyll contents (as indicated by SPAD_Leaf_), total soluble protein, and Rubisco abundance and activities. Accordingly, these traits were more highly correlated post-anthesis (Fig. 4). The correlation between flag leaf longevity (from emergence to senescence) and HI or GY (Fig. 3) is in agreement with previous observations that controlled extension of leaf life, as promoted by fungicides, provides continuous resources for grain filling, thereby increasing GY (Pepler *et al.*, 2005). Flag leaf longevity effectively increases the source strength, by prolonging the length of time for photosynthesis to contribute to grain filling. However, this is only possible when operational photosynthetic rates can be maintained at no cost to the plant or grain quality. As highlighted by Distelfeld *et al.* (2014), wheat improvement approaches should therefore accommodate the pivotal balance between nutrient-use efficiency, crop senescence, and grain quality and yield.

In-depth analysis of the comprehensive data set enabled the identification of a number of traits and cultivars of interest for breeding increased wheat yields and improving resource use efficiency in the UK (Table 3). The positive correlation between photosynthesis pre- and post-anthesis and HI suggests that combining high photosynthetic efficiency at both growth stages would be a promising strategy for increasing GY. Importantly, cultivars showing the highest net CO_2_ assimilation rates pre-anthesis did not maintain the highest rates post-anthesis, suggesting that variation exists in the photosynthetic efficiency of recently expanded versus mature flag leaves across the 64 cultivars. Gladiator, Mercato, and Zebedee achieved the highest GYs, and all three cultivars had relatively high HI. Of the three, Zebedee and Mercato had high flag leaf photosynthesis pre-anthesis, while Gladiator had high rates post-anthesis (Fig. 5).

**Table 3.** Traits and cultivars of interest for breeding increased wheat yields in the UK

Trait	Potential
Pre-anthesis *A*	Increase photosynthetic efficiency when flag leaves are most active
Post-anthesis *A*	Increase photosynthetic efficiency at a critical stage for grain filling
Light response of *A*	Improve photosynthetic radiation use efficiency
Rubisco amount	Optimize allocation of resources and N use efficiency (NUE)
**Cultivar**	**Traits/potential**
Mercato, Zebedee	High-yielding cultivar that combines high pre-anthesis *A*_400_ with high HI
Gladiator	High-yielding cultivar that combines high post- anthesis *A*_400_ with high HI
Gatsby	High photosynthetic rates with low Rubisco amounts (improve NUE?)

A major contemporary goal of agricultural research is to improve crop yields while optimizing resource use efficiency. In the present study, Rubisco was found to account for over half of the soluble leaf protein. Gatsby combined relatively high photosynthetic rates with a low Rubisco content (Table 3), both in the present (Fig. 6C) and in a previous study (PJA, unpublished results). Improving Rubisco carboxylation efficiency has the potential to sustain or improve CO_2_ assimilation while lowering resource allocation to Rubisco, which should lead to improved resource use efficiency (Carmo-Silva *et al.*, 2015). A decrease in Rubisco content of 15–20% has been predicted to result in a 10% saving in the crop’s N requirement (Reynolds *et al.*, 2012). The hypothesis that Gatsby may have increased resource use efficiency compared with cultivars that invest more on Rubisco warrants further study.

The flag leaf plays a primary role in supplying C and N to the developing ear and has long been recognized as a major contributor of photoassimilates to the grain (Waters *et al.*, 1980; Simpson *et al.*, 1983; Lopes *et al.*, 2006), with an increasing body of evidence also suggesting a significant contribution by spike photosynthesis (Araus and Tapia, 1987; Sanchez-Bragado *et al.*, 2014). Increased sink strength could also drive increased CO_2_ assimilation and, consequently, the contribution of photosynthesis to GY (Richards, 2000; Pepler *et al.*, 2005). Indeed, additional assimilatory capacity is likely to exist, albeit latent, given that photosynthetic potential tends to exceed the rates observed under field conditions (Murchie *et al.*, 2009). However, an optimal source–sink balance will by definition depend on source strength as much as sink strength. Therefore, cultivars with high flag leaf photosynthetic potential under a range of conditions, which can be sustained throughout development, will be more likely to support consistently high wheat grain yields, by enabling the fulfilment of available sink capacity.

While both total above-ground biomass and HI correlated positively with GY, straw biomass did not correlate with GY and was in fact highest in some of the low-yielding cultivars (Fig. 2), suggesting an overinvestment in leaves and stems that do not contribute to GY. Relatively high above-ground biomass has also been reported for some old wheat cultivars in the UK and worldwide (Richards, 2000). Early crop establishment can provide competitive advantage over weeds (Coleman *et al.*, 2001; Ort *et al.*, 2011), and rapid canopy closure can increase the duration of crop photosynthesis (Richards, 2000). However, early vigour is not always beneficial under favourable growth conditions, and overinvestment in leaf area can cause leaf shadowing (Evans, 1993). Thus, the optimal balance should enable rapid early development while avoiding canopy overinvestment. Accordingly, the reduced height genes (*Rht*) underpinned the outstanding wheat yield increases during the Green Revolution by promoting increased allocation of assimilates to the grain (Hedden, 2003).

In summary, this is the first study of its magnitude, in both number of field-grown wheat genotypes and phenotypic detail on photosynthesis measured at two key growth stages. The results provide strong support to previous observations that wheat flag leaf photosynthesis contributes to GY (Fischer *et al.*, 1998; Gaju *et al.*, 2016). Flag leaf longevity and flag leaf photosynthetic activity pre- and, most significantly, post-anthesis correlated with GY. To the best of our knowledge, this is also the first report linking light response of flag leaf photosynthesis to GY in field-grown wheat. That plant biomass prior to emergence of the flag leaf was not a key determinant of GY indicates that breeding of high-yielding wheat should target the operating efficiency of flag leaf photosynthesis within the canopy, under field conditions (Ort *et al.*, 2011). Measurements of flag leaf area as a proportion of total plot leaf area in future studies will enable scaling of flag leaf photosynthesis to the canopy, and prediction of its contribution to grain filling and final GY. Moreover, field trials over multiple sites and locations will enable interpretation of the findings with respect to the genotype×environment interaction, as previously described for rice (Gu *et al.*, 2014), providing a powerful tool to accelerate success in breeding for climate-resilient yield. The development of field-based high-throughput phenotyping platforms to screen for the traits of importance identified in this research will facilitate gene discovery and the speed with which these traits can be utilized in wheat breeding programmes.

## Supplementary data

Supplementary data are available at *JXB* online.

Table S1. Meteorological data for Harpenden, UK, for the period January–August 2013.

## Author contributions

Research design: ECS, PJA, SMD, TL, CAR, MAJP; research delivery: ECS, PJA, JCS; data analyses: ECS, PJA, AM; manuscript preparation: ECS, PJA, SMD, TL, CAR, MAJP.

## Supplementary Material

Supplementary Table S1

## Abbreviations:

A: net CO_2_ assimilation rate
ERYCC: Earliness and Resilience for Yield in a Changed Climate
GY: grain yield
HI: harvest index
PPFD: photosynthetic photon flux density
Q: irradiance.
