# Supplementary material for: Phenotyping of field-grown wheat in the UK highlights contribution of light response of photosynthesis and flag leaf longevity to grain yield
Source: J Exp Bot. 2017 Jun 20;68(13):3473–86. doi: 10.1093/jxb/erx169 (PMC5853948; doi:10.1093/jxb/erx169)
Supplement: Supplementary Table S1 [file erx169_suppl_supplementary_table_s1.pdf]

Supplementary data to the manuscript:

Phenotyping of field-grown wheat in the UK highlights contribution of light response of photosynthesis and flag leaf longevity to grain yield

Running title: **Flag leaf photosynthesis and wheat yield**

Elizabete Carmo-Silva, P. John Andralojc, Joanna C. Scales, Steven M Driever, Andrew Mead, Tracy Lawson, Christine A. Raines, Martin A.J. Parry

For correspondence, please contact: e.carmosilva@lancaster.ac.uk.

Table S1. Meteorological data for the wheat growing season of 2013 in Harpenden, UK. Average daily solar irradiance, total monthly sunshine, average monthly maximum and minimum air temperatures and total monthly rainfall.

Month	Sunshine (MJ m^{-2} d^{-1})	Sunshine (Hours)	Maximum temperature (°C)	Minimum temperature (°C)	Total rainfall (mm)
January	2.3 ± 0.3	45	4.9 ± 0.8	0.5 ± 0.9	63
February	4.5 ± 0.4	86	5.2 ± 0.6	0.2 ± 0.3	43
March	6.6 ± 0.6	73	5.2 ± 0.7	-0.2 ± 0.5	83
April	14.1 ± 0.9	194	12.2 ± 0.8	3.0 ± 0.7	33
May	15.5 ± 1.3	183	14.8 ± 0.5	6.1 ± 0.5	56
June	17.3 ± 1.2	185	18.0 ± 0.4	9.3 ± 0.5	25
July	20.7 ± 1.1	277	24.5 ± 0.6	12.8 ± 0.4	47
August	15.5 ± 0.8	191	22.1 ± 0.4	12.7 ± 0.4	57
